# The Prognostic and Clinical Value of CD44 in Colorectal Cancer: A Meta-Analysis

**DOI:** 10.3389/fonc.2019.00309

**Published:** 2019-04-30

**Authors:** Zhenpeng Wang, Yufei Tang, Lei Xie, Aiping Huang, Chunchun Xue, Zhen Gu, Kaiqiang Wang, Shaoqi Zong

**Affiliations:** ^1^Pain Management, Shanghai Municipal Hospital of Traditional Chinese Medicine, Shanghai University of Traditional Chinese Medicine, Shanghai, China; ^2^Shanghai Municipal Hospital of Traditional Chinese Medicine, Shanghai University of Traditional Chinese Medicine, Shanghai, China; ^3^Graduate School of Shanghai University of Traditional Chinese Medicine, Shanghai, China

**Keywords:** CD44, variant, colorectal cancer, survival, prognosis, meta-analysis

## Abstract

**Background:** CD44 is widely used as a putative cancer stem cells (CSCs) marker for colorectal cancer (CRC). However, the prognostic role of CD44 in CRC remains controversial.

**Methods:** We performed a systematic review and meta-analysis to evaluate the association of various CD44 isoforms and overall survival (OS) and clinicopathological features of CRC patients.

**Results:** A total of 48 studies were included in the meta-analysis. Total CD44 isoforms overexpression was significantly correlated with worse OS of patients with CRC (HR = 1.32, 95% CI = 1.08–1.61, *P* = 0.007). In a stratified analysis, a higher level of either CD44v6 or CD44v2 had an unfavorable impact on OS (HR_CD44v6_ = 1.50, 95% CI = 1.10–2.14, *P* = 0.010; HR_CD44v2_ = 2.93, 95% CI = 1.49–5.77, *P* = 0.002). Additionally, CD44 was shown to be associated with some clinicopathological features, such as lymph node metastasis (OR_CD44_ = 1.56, 95% CI = 1.01–2.41, *P* = 0.044; OR_CD44v6_ = 1.97, 95% CI = 1.19–3.26, *P* = 0.008; OR_Total CD44 isoforms_ = 1.57, 95% CI = 1.15–2.14, *P* = 0.004), distant metastasis (OR_CD44_ = 2.90, 95% CI = 1.08–7.83, *P* = 0.035; OR_Total CD44 isoforms_ = 1.89, 95% CI = 1.02–3.53, *P* = 0.044). Moreover, a high level of CD44 showed a possible correlation with poor differentiation (OR_Total CD44 isoforms_ = 1.44, 95% CI = 1.00–2.08, *P* = 0.051), elevated level of CD44v6 tend to be correlated with tumor size (OR = 1.71, 95% CI = 0.99–2.96, *P* = 0.056).

**Conclusions:** This meta-analysis demonstrated that CD44 overexpression might be an unfavorable prognostic factor for CRC patients and could be used to predict poor differentiation, lymph node metastasis and distant metastasis.

## Introduction

Although therapies for colorectal cancer (CRC) has improved in recent years, colorectal cancer is still the third most common cause of cancer related death worldwide (1). Metastasis are observed in 25% of patients at initial diagnosis and approximately 50% of patients will develop metastasis (2). Presently, the outcome prediction and the therapy schedule determination of CRC patients is based on the TNM classification (3). However, TNM classification cannot precisely predict the prognosis of CRC patients at an early stage, therefore, finding the bio-markers in CRC patients is very important for diagnosis and prognosis prediction.

Currently, accumulating evidence supports a hypothesis that a subpopulation of cancer cells, called cancer stem cells (CSCs) exist, which contribute to tumor initiation, recurrence and resistance to radio-or chemotherapy (4–7). Although CSCs play crucial roles in cancer initiation and progression, there is no normalized CSCs marker. It has recently been reported that CSCs markers, such as CD133, CD44, EpCAM, and ALDH1, are potential prognostic markers for various cancers (7–9). Among them, CD44 is the most common reported CSCs marker in CRC (8, 9).

CD44 is an important membrane receptor for hyaluronic acid (HA), which has been reported to activate various tumor biological behaviors, including proliferation, differentiation, invasion and motility (10–14). The alternative splicing of variable exons in the mRNA of CD44 bring about plentiful variants, including CD44v2, CD44v3, CD44v5, CD44v6 and so forth, and CD44v is only detected in some epithelial cells. Additionally, the isoform with no variable exons of CD44 is called CD44s (15), which is the smallest CD44 molecular (85–95 kDa) and expressed on vertebrate cells (15, 16). CD44s and its isoforms play a different role in cancer (16). Previous studies have reported that CD44 activates a number of signaling pathways, including the MAPK, PI3K/Akt and Wnt pathways. The activation of these pathways is linked to tumor growth, migration, EMT, chemoresistance and apoptosis resistance (17–21). Additionally, CD44 has been shown to localize MMP-9 activity to the cell surface and then enhance tumor growth and metastasis (22).

Several reports have demonstrated that the overexpression of CD44s and CD44 variants was associated with prognosis and clinicopathological features in some tumors, including CRC (23–25). However, some published studies concluded that loss of CD44 is a poor prognostic factor for CRC patients (26–28). Currently, a series of meta-analyses and published studies have proved that CD44 is a promising prognostic biomarker for head and neck cancer (23), gastric cancer (29), hepatocellular cancer (30), and other cancers (31, 32). However, there is no systematic review and meta-analysis for assessing the prognostic value of CD44 in CRC. Thus, we performed the present meta-analysis to evaluate the prognostic value of CD44 and to clarify the relationship between CD44 and clinicopathological features in patients with CRC.

## Materials and Methods

### Search Strategy

The search strategy used in the present meta-analysis was in accordance with the PRISMA statement (33). Relative studies were searched in PubMed, Embase and the Cochrane library using combination terms: (“Colorectal Neoplasm” OR “Colorectal Tumor” OR “Colorectal Carcinoma” OR “Colorectal Cancer” OR” Colonic Neoplasm” OR” Colon Cancers” OR “Colonic Cancer” OR “Rectum Cancers” OR “Rectal Tumor” OR” Rectal Cancer” OR “CRC”) [Title/abstract] AND “CD44” [Title/abstract]. In addition, we read relative review articles and manually searched relevant studies. The last search was performed on 7 November 2018.

### Inclusion Criteria

Primary studies were included under the following conditions:

(1) The study evaluated the expression level of CD44 in primary tumor tissues after surgical resection; (2) The sample size was more than 45 in the overall survival analysis; (3) A definite stage was reported; (4) The most recent and complete study was selected when the same author published several papers in the field of CD44; (5) Studies were written in English and (6) published in a peer-review journal; (7) Hazard ratio (HR) and 95% confidence intervals (CIs) were reported, or the data was sufficient to estimate the HR and 95% CIs from the survival analysis.

### Data Extraction and Management

Two authors (ZSQ and WZP) independently extracted data from each eligible article. The predefined table was used to record the baseline characteristics including the first author's name, year of publication, CD44 isoform type, nationality, sample size, detection method, HRs and 95% CIs, and method to estimate HRs (univariate and multivariate analysis), median follow-up years, and REMARK scores (Table 1). If HRs and 95% CIs were not directly reported in eligible studies, we extracted and estimated HR and 95% CIs using a method reported by Parmar et al (58, 59).

**Table 1:** Major features of included studies.

**Study**	**Country**	**Sample size**	**CD44 type**	**Detection method**	**Stage**	**HR estimation**	**Survival analysis**	**Median follow up (mouths)**	**RS**
Bhatavdekar et al. (34)	India	98	CD44	IHC	Duke's B–C	Survival curve	Univariate	43.01 (2–60)	11
Sokmen et al. (35)	Turkey	111	CD44	IHC	I–IV	Survival curve	Univariate	41.72 (1–96)	9
Ropponen et al. (36)	Finland	180	CD44s	IHC	I–IV	Survival curve	Univariate	NA (1–160)	13.5
Visca et al. (37)	Italy	100	CD44	IHC	I–III	Reported	Multivariate	36 (1–60)	12
Ngan et al. (38)	Japan	140	CD44	IHC	II–III	Reported	Multivariate	60 (0.7–150)	12
Horst et al. (39)	Germany	110	CD44	IHC	I–II	Survival curve	Univariate	94.8 (4.8–162)	12.5
Lugli et al. (27)	Switzerland	1420	CD44	IHC	I–IV	Survival curve	Univariate	NA	10
Li et al. (24)	China	57	CD44S	IHC	I–IV	Reported	Multivariate	60 (NA)	11.5
Wu et al. (25)	China	174	CD44	IHC	I–IV	Survival curve	Univariate	51.78 (8–108)	15
Seo et al. (40)	Korea	173	CD44	IHC	II–III	Reported	Univariate	43.5 (2–112)	12
Cai et al. (41)	China	117	CD44	IHC	I–IV	Reported	Multivariate	NA	12
Mohamed et al. (42)	Egypt	70	CD44	IHC	I–IV	Survival curve	Univariate	NA	11
Qu et al. (43)	China	338	CD44	IHC	II–III	Reported	Multivariate	NA	14.5
Iseki et al. (44)	Japan	49	CD44	IHC	I–IV	Survival curve	Univariate	26.7 (5.8–63.2)	14.5
Ribeiro et al. (45)	Brazil	58	CD44	IHC	IV	Reported	Multivariate	NA	13
Zavrides et al. (46)	Greece	100	CD44	IHC	I III	Calculation	Multivariate	84 (60–108)	14
Hong et al. (26)	Korea	162	CD44	IHC	I–IV	Survival curve	Univariate	83 (2–172)	13
Huh et al. (47)	Korea	74	CD44s	IHC	I–IV	Reported	Multivariate	NA	12.5
Choi et al. (48)	Korea	523	CD44s	IHC	I–IV	Survival curve	Univariate	NA	10
Saigusa et al. (49)	Japan	58	CD44	RT-PCR	II–III	Reported	Multivariate	NA	13
Fernández et al. (50)	Spain	72	CD44s	ELISA	I–IV	Survival curve	Univariate	30 (1–63)	9.5
Ropponen et al. (36)	Finland	180	CD44v6	IHC	I–IV	Reported	Multivariate	NA	13.5
Vizoso et al. (51)	Spain	105	CD44v5	ELISA	Duke's A–D	Survival curve	Univariate	15 (5–61)	16
Vizoso et al. (51)	Spain	105	CD44v6	ELISA	Duke's A–D	Reported	Multivariate	15 (5–61)	16
Saito et al. (52)	Japan	113	CD44v6	IHC	II–III	Survival curve	Univariate	NA	11
Li et al. (24)	China	57	CD44v6	IHC	Duke's A–D	Reported	Multivariate	NA	11.5
Ozawa et al. (53)	Japan	167	CD44v2	Flow cytometry	I–IV	Reported	Multivariate	NA	14
Köbel et al. (54)	Germany	145	CD44v6	IHC	I–IV	Survival curve	Univariate	NA	11
Zlobec et al. (55)	Switzerland	1236	CD44v6	IHC	I–IV	Reported	Multivariate	NA	12
Koretz et al. (56)	Netherlands	180	CD44v6	IHC	Duke's A–D	Survival curve	Univariate	70.6 (NA)	6
Jungling et al. (28)	Germany	103	CD44v6	RT-PCR	I–IV	Reported	Multivariate	57.5 (11–79)	16
Haruyama et al. (57)	Japan	63	CD44v2	IHC	Duke's B	Reported	Multivariate	65 (3–85)	13
Haruyama et al. (57)	Japan	178	CD44v6	IHC	Duke's B–C	Survival curve	Univariate	65 (3–85)	13

*RS, REMARK scores; ELISA, enzyme linked immunosorbent assay; IHC, immunohistochemistry; RT-PCR, Reverse Transcription-Polymerase Chain Reaction; multivariate, multivariate analysis; univariate, univariate analysis; Reported, HR and relative 95% CI were reported in article; Survival curve, HR and relative 95% CI were estimated from survival curves*.

### Methodological Assessment

The quality of included studies was assessed using REMARK guidelines (60). Two reviewers (TYF, WZP) evaluated the study quality and reported scores independently. Finally, all authors discussed together to reach a consensus value.

### Statistical Analysis

The individual HRs and relative 95% CIs were pooled into a summary HR and 95% CIs to assess the impact of CD44 on overall survival (OS) (61). For the measurement of the correlation of CD44 with clinicopathological parameters, odds ratios (ORs) and 95% CIs were used to estimate the effect. If HR or OR > 1 it represented a worse prognostic value of CD44 or a significant correlation between CD44 and clinicopathological features, respectively. If the 95% CI did not include the value 1, the pooled result was considered statistically significant. The heterogeneity across studies was detected using the Q test and I^2^ test, and a random-effect model (DerSimonian-Laird method) was used to calculate ORs and HRs when I^2^ was more than 50%; otherwise, a fixed-effects model (Mantel-Haenszel method) was used (62, 63). A subgroup analyses of the association between CD44 expression and prognosis were performed by CD44 types (Total CD44 isoforms, CD44, CD44v2, CD44v5, CD44v6); detection method (IHC, ELISA, RT-PCR, Flow cytometry); race (Caucasian, Asian, Black); Publication year (<2010 or ≥2010); tumor stage (I-III, I-IV, IV); univariate or multivariate analysis; REMARK scores (≤12, >12). Publication bias was assessed using Egger's test and Begg's funnel plot (64, 65). All statistical analyses were conducted using STATA version 12.0 (STATA, College Station, TX).

## Results

### Search Results

**Figure 1:**
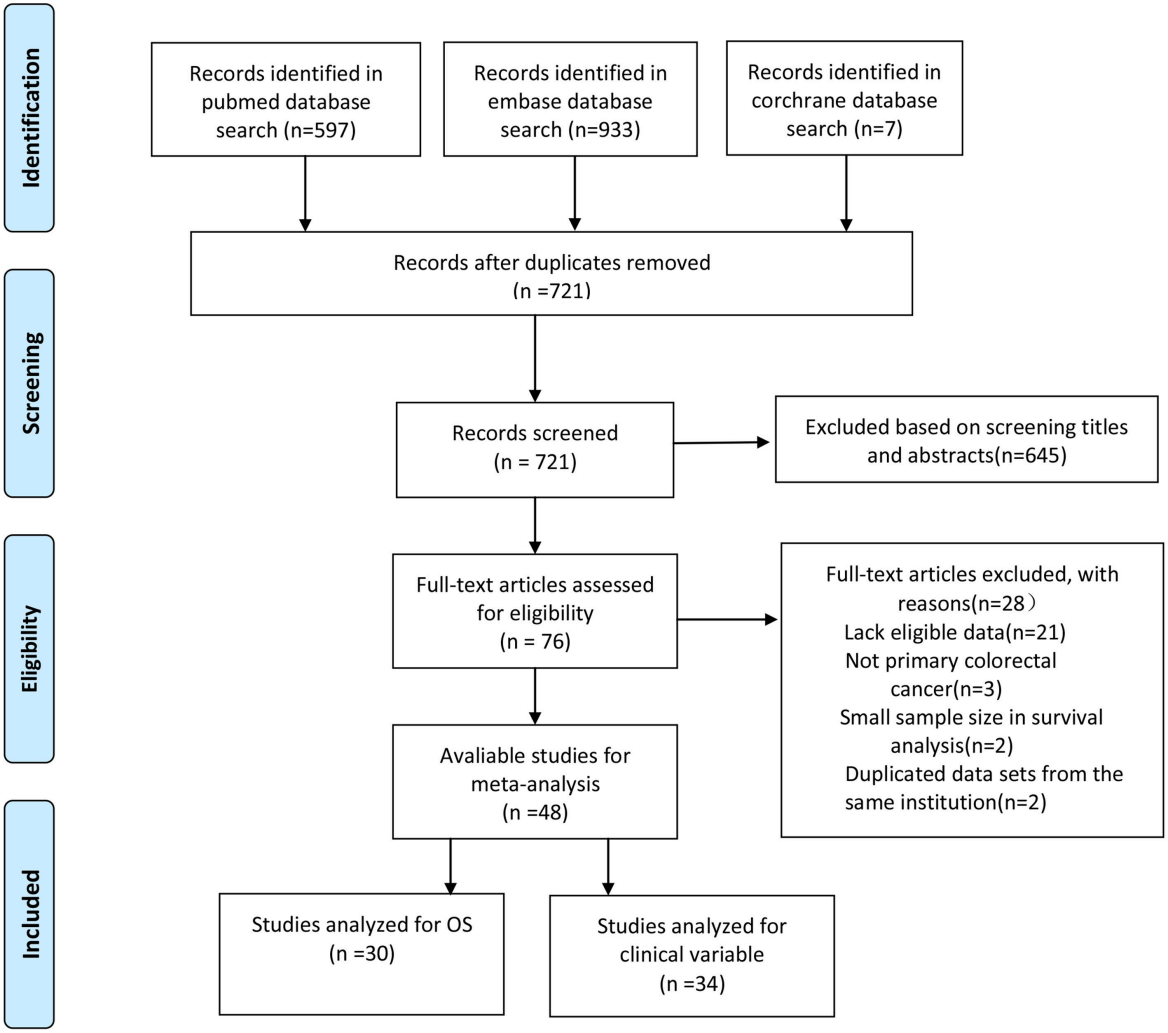
Flow diagram of study inclusion.

The literature search strategy is shown in Figure 1. A total of 48 studies were included in the final analysis. Of these studies, 30 studies that evaluated the prognostic role of CD44 in CRC patients were in accordance with the inclusion criteria (24–28, 34–57, 66). Thirty four studies reported the association of CD44 expression with clinicopathological features (24, 26, 27, 35, 39–42, 44, 46, 47, 49, 52, 54, 57, 67–84).

### Quality of Studies

Study quality was evaluated using the REMARK guidelines, the results showed that 17 studies had scores of more than 12, and the remaining 16 studies had quality scores of 12 or less.

### Study Results Report

The individual HRs and 95% CIs were obtained using a previous method (58). Sixteen studies directly reported HRs and 95% CIs. One study provided the total number of events, *P*-value and coefficient statistical value. In the remaining studies, HRs and 95% CIs were estimated using graphical survival plots.

### Study Characteristics

The characteristics of the 30 studies that evaluated the prognostic role of CD44 in patients with CRC are summarized in Table 1. Studies were mainly from Asian and European nations including Japan (*n* = 7), China (*n* = 5), Korea (*n* = 4), India (*n* = 1), Turkey (*n* = 1), Germany (*n* = 3), Greece (*n* = 1), Finland (*n* = 1), Spain (*n* = 2), Switzerland (*n* = 2), Netherlands (*n* = 1), and the remaining two studies from Egypt (*n* = 1) and Brazil (*n* = 1). The studies included mainly focused on the total CD44 (*n* = 16) more than on CD44v6 (*n* = 9) and other isoform CD44s (*n* = 5), CD44v2 (*n* = 2), CD44v5 (*n* = 1), and these studies were published between 1994 and 2018. Four detection methods including immunohistochemistry (IHC, *n* = 27), fluorescence Quantitative PCR (RT-PCR, *n* = 2), enzyme-linked immunosorbent assay (ELISA, *n* = 3) and flow cytometry (FC, *n* = 1) were applied to detect the level of CD44 (Table 1).

### Overall Survival

**Figure 2:**
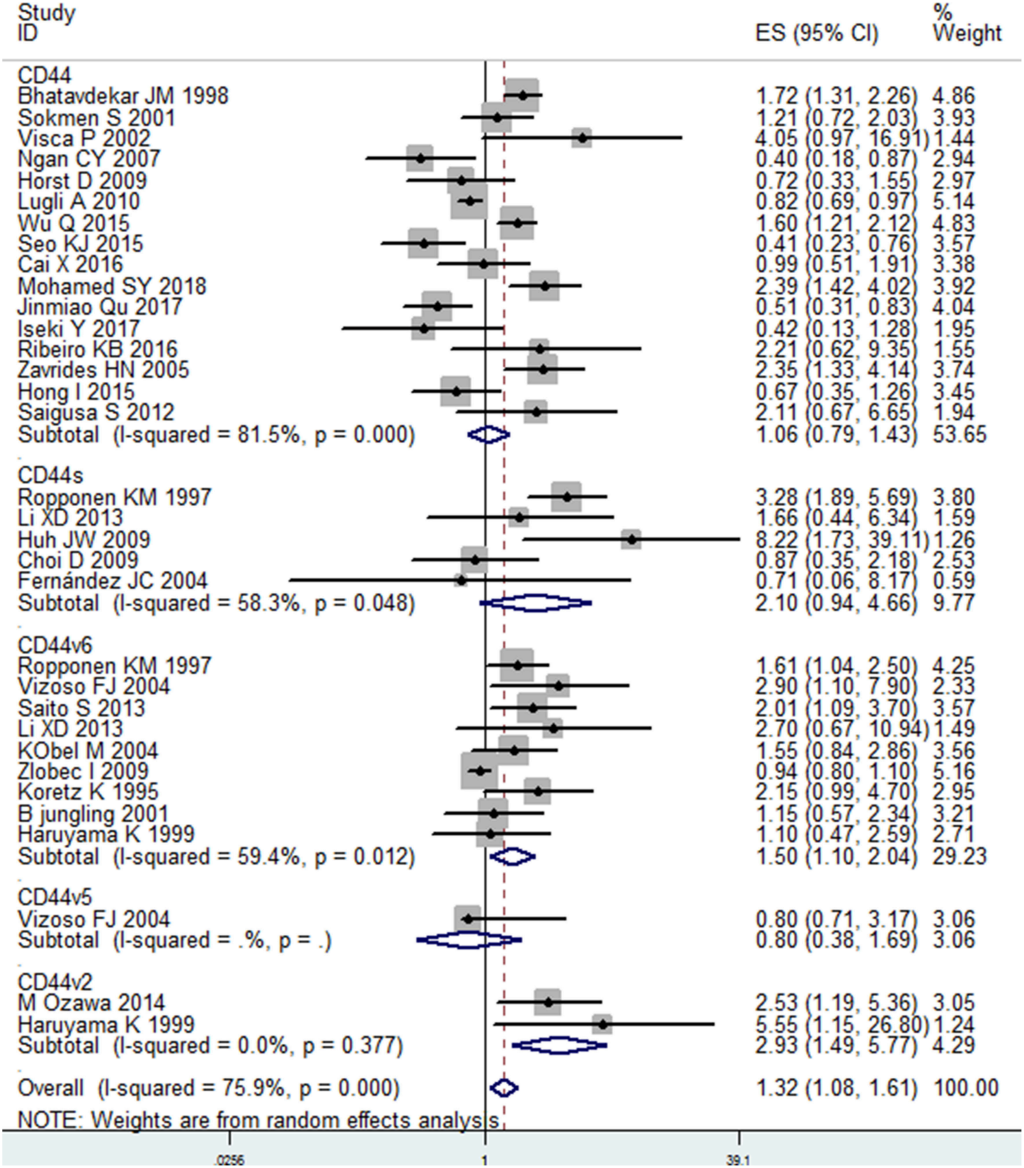
Subgroup analysis of the association between different isoforms of CD44 and OS in patients with colorectal cancer.

A total of 30 studies with 6816 patients were selected for survival analysis. CD44 overexpression was significantly associated with a worse OS (HR = 1.32, 95% CI = 1.08–1.61, *I*^2^ = 75.9%; Figure S1). The subgroup and meta-regression analyses did not find the source of heterogeneity (Table 2). However, we observed that worse OS was significantly associated with the overexpression of CD44v2 (HR = 2.93, 95% CI = 1.49–5.77, *P* = 0.002), CD44v6 (HR = 1.50, 95% CI = 1.10–2.14, *P* = 0.010) and total CD44 isoforms (HR = 1.32, 95% CI = 1.08–1.61, *P* = 0.007) (Figure 2). Additionally, a high level of CD44 was associated with worse OS for CRC patients of stage I-IV (HR = 1.42, 95% CI = 1.12–1.80, *P* = 0.001) (Figure S2). CD44 overexpression was also related significantly with OS in Caucasian patients (HR = 1.44, 95% CI = 1.13–1.85, *P* = 0.004) (Figure S3). Furthermore, the pooled HR estimate by IHC was 1.28 (95% CI = 1.03–1.60, *P* = 0.025) (Figure S4). Additionally, studies published before 2010 also showed a significant association between CD44 overexpression and OS (HR = 1.47, 95% CI = 1.13–1.91, *P* = 0.005) (Figure S5). Of note, the studies with REMARK scores of more than 12 showed worse OS (HR = 1.46, 95% CI = 1.05–2.02, *P* = 0.022) (Figure S6). We also observed that CD44 overexpression significantly correlated with OS in studies where HR was estimated by multivariate analysis (HR = 1.81, 95% CI = 1.13–2.91, *P* = 0.014) (Figure S7). These results suggested that CD44 overexpression might be a poor prognosis factor for patients of CRC.

**Table 2:** Subgroup analysis and meta-regression of the association of CD44 and overall survival of colorectal cancer patients.

**Stratified analysis**	**No. of patients**	**No. of studies**	**Model**	**Pooled HR (95%CI)**	***P***	***P_D_***	**Heterogeneity**	
							***I^2^***	***P_*H*_***
**CD44 Type**
CD44	3278	16	Random	1.06(0.79–1.43)	0.689	0.660	81.5%	<0.001
CD44s	906	5	Random	2.10(0.94–4.66)	0.069	0.179	58.3%	0.048
CD44v6	2119	9	Random	1.50(1.10–2.14)	**0.010**	0.318	59.4%	0.012
CD44v2	167	2	Fixed	2.93(1.49–5.77)	**0.002**	0.097	0	0.377
CD44v5	105	1	Fixed	080(0.38–1.69)	0.559	–	–	–
All isoforms	6816	33	Random	1.32(1.08–1.61)	**0.007**	–	75.9%	<0.001
**Method**
IHC	6202	27	Random	1.28(1.03–1.60)	**0.025**	0.823	78.8%	<0.001
RT-PCR	161	2	Fixed	1.36(0.74–2.48)	0.318	–	0	0.381
ELISA	282	3	Random	1.31(0.48–3.55)	0.594	0.876	54.2%	0.112
FC	167	1	Fixed	2.53(1.19–5.36)	0.016	0.533	–	–
**Race**
Caucasian	4303	16	Random	1.44(1.13–1.85)	**0.004**	0.073	76.7%	<0.001
Asia	2443	16	Random	1.15(0.79–1.67)	0.477	0.159	75.0%	<0.001
Black	70	1	fixed	2.39(1.42–4.02)	0.001	–	–	–
**Year**						0.069		
<2010	3803	19	Random	1.47(1.13–1.91)	**0.005**		72.2%	<0.001
≥2010	3013	14	Random	1.16(0.83–1.61)	0.393		79.0%	<0.001
**Stage**
I–III	1356	13	Random	1.15(0.76–1.73)	0.504	0.475	79.9%	<0.001
I–IV	5402	19	Random	1.42(1.12–1.80)	**0.001**	0.650	74.3%	<0.001
IV	58	1	Fixed	2.21(0.57–8.58)	0.252	–	–	–
**HR calculation**						0.141		
Univariate	3863	17	Random	1.20(0.91–1.58)	0.105		78.7%	<0.001
multivariate	2953	16	Random	1.81(1.13–2.91)	**0.014**		62.8%	0.003
**Score**						0.884		
≤12	3192	16	Random	1.20(0.93–1.55)	0.098		75.9%	<0.001
>12	3624	17	Random	1.46(1.05–2.02)	**0.022**		72.4%	<0.001

*HR, Hazard ratio; CI, confidence interval; P_D_, P for subgroup difference; P_H_, P for heterogeneity; Univariate, HRs were extracted from univariate cox proportional hazards models or survival curves; Multivariate, HRs were extracted from multivariate cox proportional hazards models; Score, REMARK scores. Bold values indicated that the result is significant or tends to be significant*.

### Clinicopathological Features

We evaluated the correlation between the overexpression of CD44 and clinicopathological features of CRC patients. The results were shown in Table 3, a higher level CD44, CD44_V_6, and total CD44 isoforms had a significant association with lymph node metastasis (OR_CD44_ = 1.56, 95% CI = 1.01–2.41, *P* = 0.044; OR_CD44v6_ = 1.97, 95% CI = 1.19–3.26, *P* = 0.008; OR _Total CD44 isoforms_ = 1.57, 95% CI = 1.15–2.14, *P* = 0.004) (Figure S10). In addition, a high level of Total CD44 isoforms tend to be associated with poor differentiation (OR = 1.44, 95% CI = 1.00–2.08, *P* = 0.051) (Figure S11). Additionally, CD44, and total CD44 isoforms overexpression was significantly correlated with distant metastasis (OR_CD44_ = 2.90, 95% CI = 1.08–7.83, *P* = 0.035; OR _Total CD44 isoforms_ = 1.89, 95% CI = 1.02–3.53, *P* = 0.044) (Figure S12). However, no significant correlation between other clinical features and CD44 exists (Table 3; Figures S8, S9, S14, S15).

**Table 3:** Meta-analysis of the association between CD44 and clinicopathological features of colorectal cancer.

**Stratification of CRC**	**No. of studies**	**Model**	**Pooled OR(95%CI)**	***P*-value**	***I^**2**^***	***P_***H***_*-value**
**LOCATION (COLON VS. RECTUM)**						
CD44	14	Random	1.09 (0.62–1.89)	0.768	79.0%	<0.001
CD44s	1	Random	4.00(1.16–13.82)	0.028	–	–
CD44v6	3	Fixed	0.82(0.51–1.32)	0.424	19.0%	0.291
CD44v2	1	Fixed	0.87(0.43–1.74)	0.690	–	–
Total	21	Random	1.12(0.73–1.71)	0.601	74.8%	<0.001
**LOCATION (RIGHT COLON VS. LEFT COLON AND RECTUM)**						
CD44	8	Random	1.17(0.63–2.17)	0.623	77.5%	<0.001
CD44V6	2	Fixed	0.80(0.40–1.59)	0.523	0.0%	0.689
Total	10	Random	1.10(0.65–1.86)	0.714	72.6%	<0.001
**LYMPH NODE METASTASIS (YES VS. NO)**						
**CD44**	18	Random	**1.56(1.01–2.41)**	**0.044**	79.1%	<0.001
CD44s	4	Random	1.10(0.36–3.33)	0.864	76.0%	0.006
**CD44v6**	8	Random	**1.97(1.19–3.26)**	**0.008**	58.5%	0.018
CD44v2	1	Fixed	0.91(0.39–2.12)	0.824	–	–
**Total**	31	Random	**1.57(1.15–2.14)**	**0.004**	74.8%	<0.001
**DIFFERENTIATION (YES VS. NO)**						
CD44	13	Random	1.32(0.82–2.12)	0.250	58.9%	0.004
CD44s	2	Fixed	1.98(0.69–5.70)	0.206	0.0%	0.431
CD44v6	5	Random	1.63(0.77–3.42)	0.199	56.3%	0.057
**Total**	21	Random	**1.44(1.00–2.08)**	**0.05**	52.5%	0.003
**T STAGE (T3/4 VS. T1/2)**						
CD44	15	Random	1.16(0.79–1.70)	0.464	54.0%	0.007
CD44v6	6	Random	0.88(0.46–1.68)	0.704	57.0%	0.040
CD44s	1	Fixed	0.35(0.07–1.84)	0.214	–	–
Total	22	Random	1.03(0.76–1.41)	0.846	51.5%	0.003
**METASTASIS (YES VS. NO)**						
**CD44**	4	Random	**2.90(1.08–7.83)**	**0.035**	77.2%	0.004
CD44s	3	Random	0.88(0.08–9.56)	0.916	73.8%	0.022
CD44v6	4	Random	1.61(0.65–4.02)	0.307	46.2%	0.134
**Total**	11	Random	**1.89 (1.02–3.53)**	**0.044**	64.1%	0.002
**TUMOR SIZE (>5 CM VS**. **≤5 CM)**						
CD44	12	Fixed	1.03(0.82–1.29)	0.788	11.5%	0.332
CD44S	2	Random	0.73(0.23–2.26)	0.581	59.0%	0.118
**CD44v6**	2	Fixed	**1.71(0.99–2.96)**	**0.056**	0.0%	0.520
Total	16	Fixed	1.08(0.88–1.32)	0.465	21.6%	0.208
**LYMPH NODE INVASION (YES VS. NO)**						
CD44	11	Random	1.54(0.85–2.78)	0.153	76.7%	<0.001
CD44S	1	Fixed	0.10(0.02–0.44)	0.002	–	–
CD44v2	1	Fixed	0.91(0.39–2.12)	0.114	–	–
CD44v6	2	Fixed	1.62(0.89–2.96)	0.824	0.0%	0.895
Total	15	Random	1.29(0.79–2.09)	0.307	74.0%	<0.001

*CRC, colorectal cancer; OR, Odds Ratio; CI, confidence interval; Total, total CD44 isoforms. Bold values indicated that the result is significant or tends to be significant*.

### Publication Bias

Egger's test and Begg's test were performed to evaluate the publication bias of OS analysis. The study of the impact CD44 overexpression on OS suggested a Begg's test score of *P* = 0.722, however, an Egger's test score of *P* = 0.062 reflected slight evidence of publication bias in the analysis. The funnel plot also revealed slight evidence of publication bias (Figure S13).

## Discussion

CD44, is a transmembrane glycoprotein and an adhesion molecule, which was first discovered on lymphocytes in 1982 (85), and is commonly accepted as a CSC marker in solid tumors (8, 86–89). In previous studies, it has been reported that CD44 is associated with various tumor biological behaviors, including proliferation, metastasis, recurrence, resistance to radio- and chemotherapy (10, 90, 91). However, a pan-CD44 antibody was used to detect the level of CD44 expression in many studies, which recognizes total CD44 variants, and therefore, these reports reveal limited knowledge about the association between specific CD44 variants and tumor progression. Currently, several studies show a different role between CD44s and CD44v. Recently, CD44s has been reported to promote EMT process, and Brown RL et al. found a shift in CD44 isoforms from CD44v to CD44s during EMT (92, 93). Additionally, Mashita et al. (94) showed that high CD44s/CD44v9 expression ratios was an independent prognosis factor in CRC. However, the CD44 variants (CD44v6 and v7/8) were up-regulated by hypoxia inducible factor (HIF) under a hypoxic condition (95). Additionally, CD44 variants were found to regulate ROS defense by stabilizing the xCT and promoting tumor growth (96). These studies showed the distinct role of CD44s and CD44v in tumor initiation and progression.

The expression of CD44 standard, v2, v3, v6, and v9 have been demonstrated in CRC patients (36, 51, 53, 55, 57). However, there are still controversies about the prognostic role of CD44s and CD44v in patients of CRC. Here, we performed a comprehensive meta-analysis to evaluate the prognostic role of various CD44 isoforms in patients with CRC. In total, 48 studies were included in our study. The results showed that a higher level of total CD44 isoforms was significantly associated with worse OS. However, a significant heterogeneity in these studies existed, thus a subgroup analysis and meta-regression analysis was performed to explore the source of heterogeneity.

Considering that various CD44 isoforms have different roles in tumor initiation and development, total CD44 isoforms, CD44s, CD44v2, CD44v6, and CD44v9 were separately analyzed, and the results showed that CD44v2 and CD44v6 was significantly associated with worse OS in CRC patients. Previous research showed that the metastatic phenotype was associated with CD44v2-10 isoform expression, especially with CD44v6 (97, 98). Additionally, a higher expression level of CD44v6 was detected in colorectal carcinoma (77, 99). However, the certain mechanism of CD44 variant isoforms in cancer development is not well-understood and further studies were needed to explore the role of CD44s and CD44v.

In addition, subgroup analysis indicated that CD44 overexpression is a poor prognosis factor in Caucasian patients (HR = 1.44; 95% CI = 1.13–1.85), while not in another race. The genetic background and environmental factors are varied in different regions, which may cause the particular characteristics of colorectal cancer in a relative human race. Moreover, a higher level of CD44 expression was significantly associated with worse OS in studies where CD44 expression was detected using immunohistochemistry staining (IHC) (HR = 1.28; 95% CI = 1.03–1.60). IHC, which is characterized by high sensibility and specificity is widely used in clinic. ELISA is used to detect CD44 protein levels in serum, however the substantial quantities of blood CD44 is not only influenced by tumor growth, but also by immune system activity (100). Additionally, RT-PCR is just used to detect the gene expression level. Therefore, the result of IHC is more convincing than other detection methods. Furthermore, subgroup analysis showed that a higher level of CD44 significantly correlated with poor survival in studies that estimated HRs using multivariate analysis (HR = 1.65; 95% CI = 1.17–2.32). These results further prove the conclusion that CD44 overexpression is a poor prognosis factor for CRC. Moreover, we stratified the variables by clinicopathological features, a higher level of total CD44 isoforms showed a significant correlation with poor differentiation (*P* = 0.048). As we know, one of the characteristics of CSCs is undifferentiated, which is in accordance with the theory that CD44 is a CSCs marker for colorectal cancer. A higher level of CD44, CD44v6 and total CD44 isoforms significantly correlated with lymph node metastasis, overexpression of CD44 and all CD44 isoforms, was found to be markedly related with distant metastasis and these results might be the reason why CD44 overexpression contributes to a poor prognosis.

There are some limitations in our meta-analysis. First, the CD44s and CD44v were identified by various methods and antibodies in different studies. Thus, CD44s and its variants were strictly defined according to the exact reactivity of antibodies. Second, we calculated HRs and 95% CI from survival curves. Third, there was significant heterogeneity in our meta-analysis, however, we minimized heterogeneity using a random effects and subgroup analysis based on various variables, such as race, publication year, detection method etc. In addition, we performed a meta-regression analysis to explore the source of heterogeneity.

A certain degree of publication bias exists in our meta-analysis. As known, studies that possess negative results and small samples are difficult to accept for publication. In addition, negative studies are more often published in a native language, however, positive studies are more likely to be published in English journals. Therefore, “negative” results should be encouraged to publish in the future. Furthermore, only fully published articles in English journals were included, while conference abstracts and unpublished studies were excluded.

## Conclusions

In summary, our meta-analysis revealed the prognostic value of CD44 expression in CRC. Specifically, positive CD44 expression was significantly correlated with poor overall survival (OS). High CD44 expression was also associated with poor differentiation, lymph node metastasis and distant metastasis, and well-designed studies are needed to confirm the findings of our meta-analysis in the future.

## Author Contributions

SZ and KW designed the study. ZW and YT performed the literature searches and assessed the quality of included studies. LX, ZG, AH, and CX analyzed the data. SZ wrote the manuscript. All authors commented on the manuscript and made the decision to submit.

### Conflict of Interest Statement

The authors declare that the research was conducted in the absence of any commercial or financial relationships that could be construed as a potential conflict of interest.
