# Supplementary material for: The Prognostic and Clinical Value of CD44 in Colorectal Cancer: A Meta-Analysis
Source: Front Oncol. 2019 Apr 30;9:309. doi: 10.3389/fonc.2019.00309 (PMC6503057; doi:10.3389/fonc.2019.00309)
Supplement: Supplementary file 1 [file Presentation_1.pdf]

Supplementary Figures Legends:

Figure s1 Meta-analysis of impact of CD44 overexpression on overall survival of patients with colorectal cancer.

Figure s2 Subgroup analysis of the association between CD44 overexpression and OS according to patients' stage.

Figure s3 Subgroup analysis of the association between CD44 overexpression and OS according to patients' race.

Figure s4 Subgroup analysis of the association between CD44 overexpression and OS according to detection methods.

Figure s5 Subgroup analysis of the association between CD44 overexpression and OS according to publication year.

Figure s6 Subgroup analysis of the association between CD44 overexpression and OS according to study quality.

Figure s7 Subgroup analysis of the association between CD44 overexpression and OS according to the HR estimation methods (univariate or multivariate analysis).

Figure s8 Meta-analysis on the relation between different isoforms of CD44 and tumor location (colon VS rectum).

Figure s9 Meta-analysis on the relation between different isoforms of CD44 and tumor location (Right colon VS Left colon and Rectum).

Figure s10 Meta-analysis on the relation between different isoforms of CD44 and lymph node metastasis.

Figure s11 Meta-analysis on the relation between different isoforms of CD44 and differentiation.

Figure s12 Meta-analysis on the relation between different isoforms of CD44 and distant metastasis.

Figure s13 Funnel plot for the evaluation of potential publication bias in the impact of CD44 overexpression on overall survival of colorectal cancer patients.

Figure s14 Meta-analysis on the relation between different isoforms of CD44 and tumor size.

Figure s15 Meta-analysis on the relation between different isoforms of CD44 and T stage.
